# Design and implementation of virtual patients for learning of clinical reasoning

**DOI:** 10.3205/zma001241

**Published:** 2019-08-15

**Authors:** Sören Huwendiek

**Affiliations:** 1 University of Bern, Medical Faculty, Institute for Medical Education, Department for Assessment and Evaluation, Bern Switzerland

**Keywords:** virtual patients, implementation, blended learning, elearning, evaluation

## Abstract

**Introduction: **Virtual Patients (VP) are electronic interactive patient cases. The aim of this PhD project was to explore how to improve the design and implementation of VPs to foster learning of clinical reasoning.

**Methods: **This PhD report is based on five consecutive studies. Using focus groups among clerkship students, we explored design features of VP. A modified Delphi study among VP experts was used to establish a VP design typology. Validity evidence was established for a questionnaire to evaluate VP design from the student perspective. In student focus groups, we explored features on how to implement VP into a clerkship. Further, we explored students’ perception of different exam formats, in an assessment of a clerkship which includes learning with VP, by focus groups, and examined whether their psychometric properties differ.

**Results: **Aspects to improve VP design:

1. using instructional design criteria such as ensuring an appropriate level of difficulty, authenticity, interactivity, feedback, and focusing on relevant learning points,
2. implementing virtual coaching on clinical reasoning into the VP, such as asking for discriminating and confirming features, and
3. providing theory-guided instruments for systematic improvements such as the developed VP typology and VP design questionnaire.

Aspects to improve VP implementation:

1. sequencing VP and other educational activities according to complexity, and aligning
2. instruction and
3. assessment with the use of VP.

**Conclusion:** Our results are in line with insights outside of VP research. Our studies demonstrate how VP can be designed, systematically further improved, and implemented to foster learning of clinical reasoning.

## Zusammenfassung

**Einführung: **Virtuelle Patienten (VP) sind elektronische interaktive Patienten. Ziel dieser PhD-Arbeit war es, zu untersuchen, wie die Gestaltung und curriculare Einbindung von VP verbessert werden kann, um das Erlernen klinischen Denkens zu unterstützen.

**Methoden: **Dieser PhD-Bericht beruht auf fünf aufeinanderfolgenden Studien. Unter Verwendung von Fokusgruppen mit Medizinstudierenden untersuchten wir Gestaltungsmerkmale VP. Eine modifizierte Delphi-Studie unter VP-Experten wurde verwendet, um eine VP-Design-Typologie zu erstellen. Ein Fragebogen wurde entwickelt und bzgl. Validitätskriterien untersucht, um die VP-Gestaltung aus studentischer Perspektive zu evaluieren. In Fokusgruppen mit Studierenden untersuchten wir, wie VP optimal in ein Curriculum eingebunden werden können. Weiterhin untersuchten wir mit Fokusgruppen die Wahrnehmung der Studierenden von zwei verschiedenen Prüfungsfragetypen, die den Lernerfolg mit VP prüften, und untersuchten, ob sich die psychometrischen Eigenschaften dieser Fragetypen voneinander unterschieden.

**Ergebnisse: **Aspekte zur Verbesserung der VP-Gestaltung:

1. Verwendung der beschriebenen Designkriterien, wie z. B. Gewährleistung eines angemessenen Schwierigkeitsgrades, Authentizität, Interaktivität, Feedback und Konzentration auf relevante Lernpunkte,
2. Umsetzung eines virtuellen Coachings bzgl. klinischem Denken im Rahmen der VP, wie z. B. Fragen nach unterscheidenden und bestätigenden Merkmalen bei Differenzialdiagnosen, und
3. Bereitstellung theorie-gestützter Instrumente für systematische Verbesserungen, wie z. B. die entwickelte VP-Typologie und der VP-Design-Fragebogen.

Aspekte der Verbesserung der curricularen Einbindung von VP:

1. Sequenz der VP und sonstiger curricularer Aktivitäten gemäß Komplexität sowie der didaktischen und inhaltlichen Abstimmung von
2. Lernen und
3. Prüfen unter Verwendung von VP.

**Schlussfolgerung: **Unsere Ergebnisse stimmen überein mit Erkenntnissen außerhalb der VP-Forschung. Unsere Studien zeigen, wie VP gestaltet, systematisch verbessert und curricular eingebunden werden können, um das Erlernen klinischen Denkens zu unterstützen.

## Introduction

Clinical reasoning is generally seen as a crucial component of nearly everything doctors do in practice [4]. It guides them to make correct diagnoses and reach appropriate treatment decisions for their patients. As such, a physician’s clinical reasoning skills are highly important and relevant for patient outcomes and patient safety [3]. However, studies have indicated that clinical reasoning is currently not well taught [7]. According to the literature, so-called Virtual Patients (VPs) are particularly suited to foster learning of clinical reasoning [2]. Virtual Patients are online cases in which the learner, takes on the role of physician, is required to make all decisions him/herself and subsequently receives feedback. Ellaway et al. [5] define VP as “interactive computer simulation[s] of real-life clinical scenarios for the purpose of medical training, education, or assessment”. Two aspects have been found to be essential regarding learning with electronic learning tools such as VPs: their design and their implementation.

Literature on how to design and implement Virtual Patients to foster clinical reasoning was lacking when we embarked on this research. Therefore, at the outset of our research project, we sought evidence on how to improve learning with VPs in order to foster clinical reasoning in medical students. The main research question was as follows:

*How can the design and implementation of Virtual Patients be improved in order to foster learning, particularly with respect to clinical reasoning?*

## Methods

Three of the five studies (studies 1,4,5) were conducted in an authentic educational practice setting, specifically a paediatric clinical clerkship. While in these three studies students in their paediatric clerkship were the main target of our investigation, in two studies (studies 2 and 3) VP experts and medical teachers, national and international, were strongly involved in the development of both the VP typology and the VP design questionnaire. Different research methods were used. In specific: 

**Study 1** explores students’ perceptions of the ideal features of Virtual Patient design to foster their learning, focusing on clinical reasoning. Fifth year students (n=104) were exposed to at least eight VPs varying in design, and discussed Virtual Patient design in focus groups [11]. 

**Study 2** describes the development of a Virtual Patient typology based on the literature, a review of existing VP systems, results from study 1, and an international consensus process among Virtual Patient experts, applying a modified Delphi technique [9]. The virtual patient expert team consisted of all six authors of this paper.

**Study 3** reports on the development and validation of a questionnaire to evaluate Virtual Patient design, drawing on published evidence, input from an international VP expert team (from eVIP project, see www.virtualpatients.eu) and including the insights gained from studies 1 and 2. Three sources of validity evidence were examined:

1. Content,
2. response process and
3. internal structure [8].

**Study 4** seeks evidence to guide the implementation of Virtual Patients. Students’ perceptions of different scenarios aimed at developing clinical reasoning skills were explored using focus groups (39 from 116 exposed students). In these scenarios, at least ten Virtual Patients were integrated with other educational activities during a paediatric clerkship [10].

**Study 5** focuses on two different types of assessment question and explores their educational impact on the learning of clinical reasoning with Virtual Patients. After students (n=377) underwent clinical clerkships and corresponding exams (n=11), they discussed in focus groups (n=8) the perception and impact of Key-Feature Problems with Long Menu (KFP) and context-rich Single Best Answer questions (crSBA). The study additionally compares the psychometric characteristics of the two formats [12].

## Results

### Study 1

Five student focus groups revealed 10 principles of VP design. To facilitate learning, Virtual Patients should be

1. relevant,
2. encompass an appropriate level of difficulty,
3. be interactive,
4. provide specific feedback,
5. utilize different, appropriate media,
6. direct students’ focus to relevant learning points,
7. foster the recapitulation of key learning points,
8. be authentic regarding the web-based interface and
9. tasks, and encompass
10. questions and explanations that enhance clinical reasoning.

After exposure to – from the student perspective – well-designed VPs, students felt very well prepared for reasoning in real patients, and better prepared than with any other previously experienced method. Students perceived the identified design principles as conducive to their learning. 

#### Study 2

We synthesised 19 factors concerning four categories identified as relevant to VP design: general (title, description, language, identifier, provenance, typical study time); educational (educational level, educational modes, coverage, objectives); instructional design (path type, user modality, media use, narrative use, interactivity use, feedback use); technical (originating system, format, integration and dependence). 

#### Study 3

The short VP design questionnaire comprises one global score and six questions distributed between three factors: authenticity of patient encounter and consultation, cognitive strategies in the consultation, coaching during consultation. Content analysis was reasonably supported by the theoretical foundation and the Virtual Patient experts (n=9) from the eVIP project [https://virtualpatients.eu/]. The think-aloud studies and the analysis of free text comments supported the questionnaire’s validity. The exploratory factor analysis, encompassing 2547 student evaluations of 78 Virtual Patients from three countries yielded a three-factor model showing a reasonable fit with the data. To reliably evaluate a Virtual Patient on all three factors, a minimum of 200 student responses are required. 

#### Study 4

The analysis of eight focus group interviews revealed six themes which were deemed by students to be important for the optimal implementation of VPs:

1. continuous and stable online access,
2. increasing complexity, adapted to students’ knowledge,
3. VP-related workload offset by elimination of other activities,
4. optimal sequencing (e.g.: lecture – 1 to 2 VP(s) – tutor-led small group discussion – real patient),
5. optimal alignment of Virtual Patients and educational activities, and
6. inclusion of VP topics in assessment.

#### Study 5

The analysis of 8 focus groups revealed four themes: Compared to the context-rich Single Best Answer format, Key-Feature Problems with Long Menu were seen as

1. more realistic,
2. more difficult and
3. more motivating for the intense study of clinical reasoning with Virtual Patients.

Moreover, overall, they

1. showed good acceptance when taking into account some preconditions like offering an additional free-text comment field in case students did not find a suitable answer in the long menu.

According to the statistical analysis, there was no difference in difficulty; however, a higher reliability (G coefficient) was found for Key-Feature Problems with Long Menu, even when corrected for testing time (reliability per testing time 1 hour: KFP 0,84 vs crSBA 0,79). The Key-Feature Problems showed higher correlations with the OSCE results (OSCE-KFP: 0,54; OSCE-crSBA: 0,41). For the study of clinical reasoning with Virtual Patients, students perceived the Key-Feature Problems as more motivating. The inclusion of Key-Feature Problems with Long Menu into summative clerkship exams seems to offer positive educational effects and no psychometric drawbacks.

## Summary and Discussion

**Studies 1-3** suggest that the following three main aspects are particularly relevant for optimising VP design to foster learning of clinical reasoning:

1. use of instructional design criteria, i.e. ensuring appropriate difficulty, authenticity, interactivity, feedback and focus on relevant learning points,
2. implementation of virtual coaching on clinical reasoning into the VP, i.e. asking for discriminating and confirming features, and
3. provision of validated instruments to enable systematic further improvements, such as the developed VP typology and VP design questionnaire.

The empirically derived VP typology provides a common reference point for studies or reports on Virtual Patients. The VP design questionnaire has the potential to provide valid information about Virtual Patient design, contingent on a large number of responses per VP.

**Studies 4-5 **suggest the following three main aspects as being especially relevant for VP implementation:

1. sequencing VPs and other educational activities, including bedside teaching with real patients, according to complexity, and
2. aligning instruction and
3. assessment with the use of VPs.

Our findings are in accordance with current theories and insights outside of VP research e.g. on instructional design [6], on how to foster learning of clinical reasoning [1], and on instructional design theories regarding curriculum development [13]. 

The main implications of our research for educational practice are as follows: To optimally foster clinical reasoning, Virtual Patients should be well designed, systematically improved through validated instruments, and well implemented.

The major strengths of this dissertation include the authentic study settings, the involvement of different stakeholders in the studies, and the combination of different research methodologies. 

Limitations of this dissertation are that three studies were done at only one centre, that it did not include a study on the longitudinal implementation of VPs, or one on the transfer of learning to real patients using objective measures. Further research is necessary to deepen the understanding of Virtual Patient design and implementation from a multicentre perspective and in different contexts. Further, the efficacy and effectiveness of VP should be investigated more in depth. This should include longitudinal studies and measurements of the impact on patient outcomes.

## A piece of advice

Ensure that you have blocked off sufficient time for your PhD work, choose your topic and supervisors wisely, and enjoy this time.

## Biographical note

Sören Huwendiek graduated from medical school at Heidelberg University in Germany, where he also worked for 10 years as a physician (finally paediatrician and paediatric rheumatologist) and as a medical educator. He gained a Masters of Medical Education degree from Bern University and this PhD in Health professions education from Maastricht University. Since 2012, he has been Head of the Department of Assessment and Evaluation of the Institute of Medical Education (IML) in Switzerland. In 2018, he was promoted to Associate Professor for Medical Education.

The defense took place at the University of Maastricht on 25 November 2016.

Supervisors: Prof. Dr. D.H.J.M Dolmans (University of Maastricht) and Prof. Dr. C.P.M. van der Vleuten (University of Maastricht). Co-supervisor: Dr. B. de Leng (University of Münster).

## Acknowledgement

My sincere gratitude goes to my great supervisors Diana Dolmans, Cees van der Vleuten and Bas de Leng from Maastricht University resp. Münster University and Burkhard Tönshoff and Georg F. Hoffmann from Heidelberg University. I also thank the many other people who contributed to and supported me in this PhD.

## Competing interests

The author declares that he has no competing interests.
